# Supplementary material for: Influence of Executive Function Training on BMI, Food Choice, and Cognition in Children with Obesity: Results from the TOuCH Study
Source: Brain Sci. 2023 Feb 17;13(2):346. doi: 10.3390/brainsci13020346 (PMC9954074; doi:10.3390/brainsci13020346)
Supplement: Supplementary file 1 [file brainsci-13-00346-s001.zip › brainsci-2088635-supplementary.pdf]

Table S1. Main outcomes at baseline and group comparison (PP analyses)

	Experimental group			Control group			Group comparison	
**Weight and diet**	**N**	**Mean**	**SD**	**N**	**Mean**	**SD**	***T/U***	**Sig.**
BMI (kg/m²)	19	29.29	3.18	17	29.70	4.22		
WC (cm)	19	91.94	9.18	16	92.53	9.57	146.500	.855
Kidmed (raw score)	18	7.11	1.86	17	6.71	2.02	133.000	.500
**Cognitive tests (raw score)**								
**WISC-V**								
Digit span forward	19	5.63	1.38	17	5.29	.77	152.5	.75
Digit span backward	19	4.42	.84	17	4.18	1.02	129.0	.28
**WNV**								
Spatial span forward	19	5.74	1.15	17	5.65	1.22	143.0	.54
Spatial span backward	19	5.21	.71	17	5.29	1.21	160.5	.97
**ToL**								
Total move	19	38.26	15.19	17	36.41	24.21	128.0	.29
Total time	19	304.16	117.94	17	302.18	207.86	132.0	.35
**CCTT**								
Part I	19	25.89	11.16	17	24.29	9.98	-.45	.65
Part II	19	49.16	14.24	17	49.35	11.94	.04	.96
**FDT**								
Reading	19	25.79	4.21	17	26.29	4.40	147.5	.66
Counting	19	33.05	5.67	17	35.06	6.12	1.02	.31
Choosing	19	56.42	11.99	17	56.76	7.60	143.0	.62
Shifting	19	62.89	12.77	17	63.59	10.60	146.0	.62
**CPT (T-score)**								
Detectability	19	55.00	9.32	17	56.94	9.22	140.0	.50
Omissions	19	49.32	6.79	17	54.47	9.25	109.5	.10
Hit Reaction Time	19	47.42	6.44	17	48.76	7.09	.60	.56
**N-back**								
Correct responses 0-back	19	5.05	.71	17	5.26	.73	129.5	.30
Correct responses 1-back	19	5.08	1.00	17	5.00	1.16	159.5	.95
Correct responses 2-back	19	4.26	1.33	17	4.06	1.58	153.0	.79
Correct responses 3-back	19	3.08	.93	17	3,09	1,08	.03	.98
**Go-No Go**								
Correct responses	19	214.58	20.88	16	210.00	30.74	143.0	.77
Commissions	19	32.89	19.42	16	33.25	13.62	138.5	.66
**Rating scales (raw score)**								
BRIEF								
Cognitive	19	52.74	13.589	17	52.65	13.65	-.02	.98
Emotional	19	25.68	5.86	17	26.53	6.29	.42	.68
Behavioral	19	19.16	5.98	17	18.71	3.97	-.26	.79
PedsQl								
Child's total score	19	76.72	11.94	17	77.94	12.78	.30	.76
Parents' total score	19	73.92	11.46	17	72.72	16.35	-.26	.80
SPPC								
Global self-esteem	18	20.50	3.70	15	18.93	4.98	112.0	.40
Social support	18	21.24	2.28	14	21.13	2.24	-.138	.89
CBCL								
Internalizing symptoms	19	7.53	4.11	17	11.30	5.73	2.28	**.03**
Externalizing symptoms	19	7.53	6.25	17	8.18	4.45	139.0	.48

Note. Bold p-values indicate statistical significance ($p < 0.05$).

Table S2. Baseline characteristics and group comparisons for all randomized participants (ITT)

	Experimental group			Control group			Group comparison	
	**N**	**Mean**	**SD**	**N**	**Mean**	**SD**	***T/U***	**Sig.**
Age (years)	25	10.40	1.04	19	10.79	.98	187.500	.213
BMI (percentile)	25	98.54	.78	19	98.59	.76	.221	.826
Physical activity (hours)	25	2.19	1.93	19	2.87	4.25	235.500	.961
Daily screen time(hours)	25	2.55	1.35	19	1.71	0.67	142.000	.022
Visual IQ (scalar score)	25	9.84	2.58	19	10.21	2.07	224.500	.756
Verbal IQ (scalar score)	25	11.16	2.70	19	10.53	2.25	-.827	.413
Motivation for treatment	23	6.26	.752	18	6.56	.783	157.000	.142
		**N**			**N**		**X^2**	**Sig.**
Sex (F:M)		10:15			8:11		0.020	.888
Handedness (R:L)		21:4			17:2		-	.684†
Economic income (I:II:III:IV:V)		7:8:2:2:4			3:4:5:4:0		7.294	.115††
Hospital (CST:SJD)		19:6			12:7		.855	.355

Note.†Fisher exact test††Freeman-Halton Exact Test

Table S3. Main outcomes at baseline and group comparison for all randomized participants (ITT)

	Experimental group			Control group			Group comparison	
**Weight and diet**	**N**	**Mean**	**SD**	**N**	**Mean**	**SD**	***T/U***	**Sig.**
BMI (kg/m^2)	25	28.97	3.16	19	30.10	4.17	203.500	.420
WC (cm)	25	91.06	9.86	18	94.70	11.01	1.137	.262
Kidmed (raw score)	24	7.04	1.99	19	6.68	1.92	196.000	.424
**Cognitive tests (raw score)**								
**WISC-V**								
Digit span forward	25	5.52	1.26	19	5.26	.73	226.000	.755
Digit span backward	25	4.32	.80	19	4.21	.98	209.500	.479
Digit span sequencing	25	5.48	1.05	19	5.05	.78	187.500	.168
**WNV**								
Spatial span forward	25	5.60	1.16	19	5.74	1.20	233.000	.912
Spatial span backward	25	5.20	1.00	19	5.37	1.17	226.000	.774
**ToL**								
Total move	25	37.40	15.28	19	35.42	23.55	188.500	.245
Total time	25	314.92	123.43	19	298.84	196.22	189.000	.250
**CCTT**								
Part I-seconds	25	26.64	10.44	19	26.79	14.82	215.500	.602
Part II-seconds	25	51.76	15.11	19	49.89	11.39	-.449	.656
**FDT**								
Reading	25	26.56	4.26	19	26.11	4.27	-.350	.728
Counting	25	34.40	6.12	19	35.32	6.96	.463	.646
Choosing	25	57.12	10.96	19	57.37	7.92	224.000	.749
Shifting	25	65.12	14.05	19	64.68	11.14	-.111	.912
**CPT (T-score)**								
Detectability	25	55.76	8.64	19	57.37	8.81	207.000	.469
Omissions	25	49.92	6.99	19	53.95	8.94	180.500	.176
Hit Reaction Time	25	48.68	7.49	19	48.89	7.48	237.000	.991
**N-back**								
Correct responses 0-back	25	5.12	.79	19	5.34	.73	197.500	.329
Correct responses 1-back	25	5.00	1.19	19	4.97	1.17	236.000	.971
Correct responses 2-back	25	4.02	1.40	19	4.16	1.52	220.000	.676
Correct responses 3-back	25	3.00	.96	19	3.18	1.07	.601	.551
**Go-No Go**								
Correct responses	25	213.48	21.16	18	210.72	29.27	218.00	.863
Commissions	25	33.56	18.36	18	33.78	13.42	204.500	.614
**Rating scales (raw score)**								
BRIEF								
Cognitive	25	51.44	13.73	19	52.68	12.91	216.500	.618
Emotional	25	25.44	5.77	19	26.32	6.07	.487	.629
Behavioral	25	18.92	5.87	19	18.26	3.97	236.000	.972
PedsQl								
Child's total score	25	75.88	12.10	19	76.77	12.61	.237	.814

Parents' total score	25	74.45	12.20	19	72.20	15.63	-.537	.594
SPPC								
Global self-esteem	24	19.38	4.63	17	18.76	4.76	187.000	.650
Social support	24	20.55	3.04	16	20.42	2.87	186.000	.868
CBCL								
Internalizing symptoms	25	8.60	4.89	19	10.53	5.88	191.000	.269
Externalizing symptoms	25	7.48	5.81	19	7.47	4.69	229.000	.840

Note. Statistical significance is set at $p < 0.05$ level.

Table S4. EF training effects on primary outcomes: (a) cognition and anthropometric pre-post measures and (b) evolution of food-choice across the 6-week training period (ITT)

					Main effects				Group by time interaction	
**(a) Pre-post primary outcomes**		**n**	**Pre-test Mean (SD)**	**Post-test Mean (SD)**	**Group F (df); p**	**η_p^2**	**Time F (df); p**	**η_p^2**	**F (df); p**	**η_p^2**
Cognition (z score)	EG	25	.01 (.462)	.05 (.504)	$F_{(1,42)}$= .236; .630	.006	$F_{(1,42)}$= .015; .902	.000	$F_{(1,42)}$= .787; .380	.018
	CG	19	-.01 (.421)	-.06 (.435)						
BMI	EG	25	28.97 (3.16)	28.48 (3.07)	$F_{(1,42)}$= 1.20; .280	.028	$F_{(1,42)}$= 11.50; **.002**	.215	$F_{(1,42)}$= .16; .689	.004
	CG	19	30.10 (4.17)	29.71 (3.99)						
WC	EG	25	91.06 (9.86)	89.21 (8.00)	$F_{(1,41)}$= 1.92; .173	.045	$F_{(1,41)}$= 6.92; **.012**	.144	$F_{(1,41)}$= 1.14; .291	.027
	CG	18	94.69 (11.01)	93.42 (10.87)						
Kidmed Food	EG	21	7.04 (1.99)	6.52 (2.36)	$F_{(1,36)}$= .05; .816	.001	$F_{(1,36)}$= .06; .804	.002	$F_{(1,36)}$= .362; .551	.010
	CG	17	6.68 (1.92)	6.59 (2.06)						

**(b) 6-week training primary outcomes[†]**		**Fixed effects**					**Random effects**				**Model fit**
		**Est/Beta**	**SE**		**t**	**p**	**Param.**	**Covariance**	**SE**	**Sig.**	**AIC/BIC**
Kidmed Modified[††]	Intercept	4.299	.537	3.203 to 5.396	8.012	.000	Residual	1.843	.256	.000	655.606/ 668.054
	Group	-.581	.904	-2.426 to 1.264	-.642	.525	Intercept + Time				
	Time	-.732	.412	-1.579 to .114	-1.766	.087	[subject] UN (1,1)	4.207	1.628	.010	
	Group x time	.118	.685	-1.285 to 1.521	.172	.864	UN (2,1)	-2.177	1.165	.062	
							UN (2,2)	1.378	.972	.156	

Note. [†]Mixed-effects model parameters for food-choice. [††] Scores could go from 0 to 15, larger scores mean better food choice. *F*, mixed ANOVA. Bold values indicate statistical significance ($p < 0.05$).

Abbreviations. BMI = body mass index; CG = control group; EG = experimental group; WC = waist circumference.

Table S5. EF training effects on secondary outcomes: post-hoc analyses of cognitive subdomains (ITT)

					**Main effects**				**Group by time interaction**	
		**n**	**Pre-test Mean (SD)**	**Post-test Mean (SD)**	**Group F (df); p**	**η_p^2**	**Time F (df); p**	**η_p^2**	**F (df); p**	**η_p^2**
**Cognitive domains**										
**Attention and speed**	EG	25	5.52 (1.26)	5.56 (.92)	$F_{(1,42)}$= .35; .560	.008	$F_{(1,42)}$= 1.08; .305	.025	$F_{(1,42)}$= .584; .449	.014
WISC-V Digit Span	CG	19	5.26 (.73)	5.53 (.61)						
Forward	EG	25	5.60 (1.16)	6.40 (.96)	$F_{(1,42)}$= .16; .695	.004	$F_{(1,42)}$= 8.81; **.005**	.173	$F_{(1,42)}$= 1.66; .205	.038
WNV Spatial Span	CG	19	5.74 (1.20)	6.05 (.97)						
Forward	EG	25	26.64 (10.44)	23.12 (9.13)	$F_{(1,42)}$= .00; .997	.000	$F_{(1,42)}$= 5.36; **.026**	.113	$F_{(1,42)}$= .01; .920	.000
CCTT Part I Time	CG	19	26.79 (14.82)	22.95 (10.08)						
	EG	25	26.56 (4.26)	25.72 (4.60)	$F_{(1,42)}$= .00; .979	.000	$F_{(1,42)}$= .61; .440	.014	$F_{(1,42)}$= .61; .440	.014
FDT Reading Time	CG	19	26.11 (4.27)	26.11 (5.21)						
	EG	25	34.40 (6.12)	32.24 (5.42)	$F_{(1,42)}$= .12; .734	.003	$F_{(1,42)}$= 17.18; **.000***	.290	$F_{(1,42)}$= .28; .601	.007
FDT Counting Time	CG	19	35.32 (6.97)	32.53 (6.03)						
	EG	25	55.76 (8.64)	55.04 (8.23)	$F_{(1,42)}$= .28; .601	.007	$F_{(1,42)}$= 1.56; .219	.036	$F_{(1,42)}$= .12; .734	.003
CPT Detectability	CG	19	57.37 (8.81)	56.11 (9.38)						
	EG	25	49.92 (6.99)	51.80 (7.93)	$F_{(1,42)}$= 2.94; .094	.065	$F_{(1,42)}$= 2.45; .125	.055	$F_{(1,42)}$= .032; .858	.001
CPT Omissions	CG	19	53.95 (8.94)	56.32 (13.31)						
	EG	25	48.68 (7.49)	49.84 (8.02)	$F_{(1,42)}$= .59; .446	.014	$F_{(1,42)}$= 7.46; **.009**	.151	$F_{(1,42)}$= 2.61; .113	.059
CPT Reaction Time	CG	19	48.89 (7.48)	53.42 (12.00)						
**Working Memory**										
WISC-V Digit Span	EG	25	4.32 (.80)	4.48 (.92)	$F_{(1,42)}$= .75; .391	.018	$F_{(1,42)}$= .24; .630	.006	$F_{(1,42)}$= .24; .630	.006
Backward	CG	19	4.21 (.98)	4.21 (.92)						

WNV Spatial Span Backward	EG	25	5.20 (1.00)	5.80 (1.32)	$F_{(1,42)}$= .21; .651	.005	$F_{(1,42)}$= 2.33; .134	.053	$F_{(1,42)}$= 2.33; .134	.053
	CG	19	5.37 (1.17)	5.37 (1.07)						
N back (1-back)	EG	25	5.00 (1.19)	4.74 (1.33)	$F_{(1,42)}$= .07; .792	.002	$F_{(1,42)}$= .76; .388	.018	$F_{(1,42)}$= .51; .480	.012
	CG	19	4.97 (1.17)	4.95 (1.26)						
N back (2-back)	EG	25	4.02 (1.40)	4.18 (1.84)	$F_{(1,42)}$= 1.26; .268	.029	$F_{(1,42)}$= 5.17; **.028**	.110	$F_{(1,42)}$= 2.27; .139	.051
	CG	19	4.16 (1.52)	4.95 (.98)						
N back (3-back)	EG	25	3.00 (.96)	3.00 (1.35)	$F_{(1,42)}$= .76; .387	.018	$F_{(1,42)}$= .11; .741	.003	$F_{(1,42)}$= .11; .741	.003
	CG	19	3.18 (1.07)	3.32 (1.13)						
**Cognitive flexibility**										
CCTT Part II Time	EG	25	51.76 (15.11)	43.24 (10.54)	$F_{(1,42)}$= .02; .899	.000	$F_{(1,42)}$= 15.67; **.000***	.272	$F_{(1,42)}$= .68; .414	.016
	CG	19	49.89 (11.39)	44.32 (7.92)						
FDT Shifting Time	EG	25	65.12 (14.05)	56.80 (13.79)	$F_{(1,42)}$= .04; .843	.001	$F_{(1,42)}$= 83.64; **.000***	.666	$F_{(1,42)}$= .11; .741	.003
	CG	19	64.68 (11.14)	55.74 (10.84)						
**Inhibitory control**										
FDT Choosing Time	EG	25	57.12 (10.96)	51.92 (10.00)	$F_{(1,42)}$= .00; .950	.000	$F_{(1,42)}$= 38.25; **.000***	.477	$F_{(1,42)}$= .22; .642	.005
	CG	19	57.37 (7.92)	51.32 (9.55)						
GNG Correct responses	EG	25	213.48 (21.16)	223.40 (18.41)	$F_{(1,40)}$= .83; .369	.020	$F_{(1,40)}$= 5.62; **.023**	.123	$F_{(1,40)}$= .26; .613	.006
	CG	18	210.72 (29.27)	214.83 (18.34)						
GNG Commissions	EG	25	33.56 (18.36)	25.32 (13.61)	$F_{(1,40)}$= .15; .704	.004	$F_{(1,40)}$= 14.47; **.000***	.266	$F_{(1,40)}$= .30; .589	.007
	CG	18	33.78 (13.42)	29.78 (16.01)						
**Planning**										
ToL Total move	EG	25	37.40 (15.28)	31.12 (14.10)	$F_{(1,42)}$= .09; .771	.002	$F_{(1,42)}$= 2.78; .103	.062	$F_{(1,42)}$= .08; .781	.002
	CG	19	35.42 (23.55)	30.95 (8.43)						
ToL Total time	EG	25	314.92 (123.43)	232.76 (106.64)	$F_{(1,42)}$= .07; .788	.002	$F_{(1,42)}$= 12.30; **.001***	.227	$F_{(1,42)}$= .10; .750	.002
	CG	19	298.84 (196.22)	230.474 (84.86)						

Note. F, mixed ANOVA. Bold values indicate statistical significance ($p < 0.05$) and * indicates Bonferroni-adjusted statistical significance ($p<.003$).

Abbreviations. CCTT = Children's Colour Trail Test; CG = control group; CPT3 = Conners' Continuous Performance Test 3; EG = experimental group; FDT = Five Digits Test; GNG: Go-no Go; ToL = Tower of London; WISC-V = Wechsler Intelligence Scale for Children (5th ed.); WNV = Weschler non-verbal.

Table S6. EF training effects on secondary outcomes: (a) post-hoc analyses of rating scales and (b) evolution of healthy habits across the 6-week training period (ITT)

					Main effects				Group by time interaction	
					Group		Time			
**(a) Rating scales (raw score)**		**n**	**Pre-test**	**Post-test**	**F (df); p**	**η_p^2**	**F (df); p**	**η_p^2**	**F (df); p**	**η_p^2**
BRIEF-2	Cognitive	24	51.44 (13.73)	50.54 (13.89)	$F_{(1,41)}$= .11; .742	.003	$F_{(1,41)}$= 8.54; **.006**	.172	$F_{(1,41)}$= 3.16; .083	.072
		19	52.68 (12.91)	47.21 (10.21)						
	Emotional	24	25.44 (5.77)	24.96 (5.99)	$F_{(1,41)}$= .08; .776	.002	$F_{(1,41)}$= 4.86; **.033**	.106	$F_{(1,41)}$= 2.24; .142	.052
		19	26.32 (6.07)	23.26 (5.57)						
	Behavioral	24	18.92 (5.87)	17.79 (5.53)	$F_{(1,41)}$= .29; .594	.007	$F_{(1,41)}$= 3.97; .053	.088	$F_{(1,41)}$= .00; .965	.000
		19	18.26 (3.97)	17.00 (3.96)						
PedsQl	Child's total score	25	75.88 (12.10)	80.13 (10.61)	$F_{(1,42)}$= .03; .863	.001	$F_{(1,42)}$= 6.35; **.016**	.131	$F_{(1,42)}$= .05; .822	.001
		19	76.77 (12.61)	80.32 (10.16)						
	Parents' total score	25	74.45 (12.20)	76.96 (14.16)	$F_{(1,42)}$= .60; .441	.014	$F_{(1,42)}$= .58; .452	.014	$F_{(1,42)}$= .17; .685	.004
		19	72.20 (15.63)	72.95 (18.35)						
SPPC	Self-esteem	22	19.38 (4.63)	21.26 (2.54)	$F_{(1,37)}$= .63; .433	.017	$F_{(1,37)}$= 7.15; **.011**	.162	$F_{(1,37)}$= .28; .598	.008
		17	18.76 (4.76)	21.16 (3.52)						
	Social support	22	20.55 (3.04)	21.25 (3.07)	$F_{(1,36)}$= .02; .889	.001	$F_{(1,36)}$= 8.80; **.005**	.196	$F_{(1,36)}$= .04; .835	.001
		16	20.42 (2.87)	21.08 (1.92)						
CBCL	Internalizing symptoms	24	8.60 (4.89)	6.92 (4.61)	$F_{(1,41)}$= 1.92; .173	.045	$F_{(1,41)}$= 4.74; **.035**	.104	$F_{(1,41)}$= .08; .774	.002
		19	10.53 (5.88)	8.95 (7.55)						
	Externalizing symptoms	24	7.48 (5.81)	7.50 (7.09)	$F_{(1,41)}$= .12; .734	.003	$F_{(1,41)}$= .75; .391	.018	$F_{(1,41)}$= .64; .428	.015
		19	7.47 (4.69)	6.42 (4.89)						

**(b) 6-week training secondary outcomes**[†]		**Fixed effects**					**Random effects**					**Model fit**
		**Est/Beta**	**SE**	**95% CI**	**t**	**p**	**Param.**		**Covariance**	**SE**	**Sig.**	**AIC/BIC**
Daily steeps	Intercept	13242.65	744.07	11733.92 to 14751.38	17.80	.000	Residual		4251008.03	522396.46	.000	3898.64/ 3911.99
	Group	-1385.67	1550.65	-3716.60 to 945.25	-1.20	.236	Intercept + Time					
	Time	-265.15	502.15	-1287.02 to 756.71	-.53	.601	[subject]	UN (1,1)	8815250.90	3127819.33	.005	
	Group x time	317.47	799.06	-1306.37 to 1941.24	.40	.694		UN (2,1)	794953.69	1610874.75	.622	
								UN (2,2)	1248415.09	1468215.27	.395	
	Intercept	493.76	7.97	477.61 to 509.91	61.98	.000	Residual		786.33	100.52	.000	

Sleep time (min.)	Group	-6.62	12.43	-31.80 to 18.56	-.53	.597	Intercept + Time				1918.52/1931.57
	Time	-17.84	6.77	-31.58 to -4.09	-2.63	**.012**	[subject] UN (1,1)	671.39	356.72	.060	
	Group x time	-.29	10.53	-21.65 to 21.08	-.03	.979	UN (2,1)	-134.11	255.19	.599	
							UN (2,2)	145.58	244.09	.551	

Note. †Mixed-effects model parameters for physical activity and sleep patterns. *F*, mixed ANOVA. Bold values indicate statistical significance ($p < 0.05$) and * indicates Bonferroni-adjusted statistical significance ($p<.005$).

Abbreviations. BRIEF-2 = Behaviour Rating Inventory of Executive Function 2; CBCL = Child Behaviour Checklist; PedsQl = Pediatric Quality of Life Inventory; SPPC = Self-Perception and Social Support Profile for Children.

Table S7. Descriptive data at each time point for diet, physical activity and sleep patterns (PP analyses)

		**Week 1**		**Week 2**		**Week 3**		**Week 4**		**Week 5**		**Week 6**	
		**n**	**Mean (SD)**	**n**	**Mean (SD)**	**n**	**Mean (SD)**	**n**	**Mean (SD)**	**n**	**Mean (SD)**	**n**	**Mean (SD)**
**Diet (modified Kidmed†)**	**EG**	16	4.48 (2.13)	18	3.92 (1.73)	17	4.13 (1.91)	14	3.75 (1.60)	16	2.95 (1.30)	16	3.36 (1.73)
	**CG**	7	3.02 (2.98)	10	4.06 (2.35)	10	3.56 (1.99)	9	2.85 (1.68)	11	3.59 (2.01)	10	2.73 (1.95)
**Daily steeps**	**EG**	18	13847.79 (3969.13)	16	13131.07 (2449.42)	16	13439.33 (3464.28)	19	13558.06 (5126.98)	19	12878.66 (4884.22)	16	14111.48 (3454.44)
	**CG**	13	11245.51 (4200.68)	12	12355 (2722.28)	13	12420.53 (2160.02)	14	12046.35 (3904.55)	15	12577.92 (3643.32)	13	11657.85 (4223.55)
**Sleep time (min.)**	**EG**	18	487.53 (42.59)	16	482.61 (38.81)	15	481.57 (30.12)	18	483.21 (30.79)	17	474.58 (22.08)	15	460.65 (37.04)
	**CG**	14	493.10 (30.20)	12	483.32 (42.15)	12	475.62 (45.09)	11	466.02 (46.85)	14	469.15 (33.78)	13	468.10 (42.09)

Note.†Scores could go from 0 to 15, larger scores mean better food choice.

Table S8. Descriptive data at each time point for diet, physical activity and sleep patterns (ITT)

		Week 1		Week 2		Week 3		Week 4		Week 5		Week 6	
		**n**	**Mean (SD)**	**n**	**Mean (SD)**	**n**	**Mean (SD)**	**n**	**Mean (SD)**	**n**	**Mean (SD)**	**n**	**Mean (SD)**
**Diet (modified Kidmed[†])**	**EG**	17	4.53 (2.07)	21	3.69 (1.75)	20	3.87 (1.93)	16	3.79 (1.53)	17	3.08 (1.37)	16	3.36 (1.73)
	**CG**	8	3.17 (2.79)	11	3.87 (2.31)	11	3.63 (1.89)	10	2.87 (1.59)	12	3.36 (2.07)	11	2.75 (1.85)
**Daily steeps**	**EG**	21	13791.25 (3941.37)	20	13215.52 (3522.52)	21	13184.62 (3615.49)	22	13162.33 (4905.59)	23	12861.92 (4765.99)	18	14152.99 (3576.66)
	**CG**	15	11144.38 (3898.31)	14	12390.69 (2521.58)	14	12332.63 (2101.18)	15	12143.46 (3781.27)	16	12824.48 (3655.35)	13	11657.85 (4223.55)
**Sleep time (min.)**	**EG**	21	485.49 (39.72)	20	483.15 (38.83)	19	480.69 (26.68)	20	479.29 (31.53)	20	473.23 (25.16)	17	461.41 (35.74)
	**CG**	14	493.10 (30.20)	14	475.50 (43.59)	13	471.62 (45.52)	12	458.20 (52.25)	14	469.15 (33.78)	13	468.10 (42.09)

Note.[†]Scores could go from 0 to 15, larger scores mean better food choice.
